# Targeted Ablation of miR-21 Decreases Murine Eosinophil Progenitor Cell Growth

**DOI:** 10.1371/journal.pone.0059397

**Published:** 2013-03-22

**Authors:** Thomas X. Lu, Eun-Jin Lim, Svetlana Itskovich, John A. Besse, Andrew J. Plassard, Melissa K. Mingler, Joelle A. Rothenberg, Patricia C. Fulkerson, Bruce J. Aronow, Marc E. Rothenberg

**Affiliations:** 1 Division of Allergy and Immunology, Department of Pediatrics, Cincinnati Children’s Hospital Medical Center, University of Cincinnati College of Medicine, Cincinnati, Ohio, United States of America; 2 Medical Scientist Training Program, Cincinnati Children’s Hospital Medical Center, University of Cincinnati College of Medicine, Cincinnati, Ohio, United States of America; 3 Division of Biomedical Informatics, Department of Pediatrics, Cincinnati Children’s Hospital Medical Center, University of Cincinnati College of Medicine, Cincinnati, Ohio, United States of America; University of Frankfurt - University Hospital Frankfurt, Germany

## Abstract

MiR-21 is one of the most up-regulated miRNAs in multiple allergic diseases associated with eosinophilia and has been shown to positively correlate with eosinophil levels. Herein, we show that miR-21 is up-regulated during IL-5-driven eosinophil differentiation from progenitor cells *in vitro*. Targeted ablation of miR-21 leads to reduced eosinophil progenitor cell growth. Furthermore, miR-21^−/−^ eosinophil progenitor cells have increased apoptosis as indicated by increased levels of annexin V positivity compared to miR-21^+/+^ eosinophil progenitor cells. Indeed, miR-21^−/−^ mice have reduced blood eosinophil levels *in vivo* and reduced eosinophil colony forming unit capacity in the bone marrow. Using gene expression microarray analysis, we identified dysregulation of genes involved in cell proliferation (e,g, *Ms4a3*, *Grb7*), cell cycle and immune response as the most significant pathways affected by miR-21 in eosinophil progenitors. These results demonstrate that miR-21 can regulate the development of eosinophils by influencing eosinophil progenitor cell growth. Our findings have identified one of the first miRNAs with a role in regulating eosinophil development.

## Introduction

Eosinophils are involved in a variety of diseases including hypereosinophilic syndrome, eosinophilic gastrointestinal disorders, asthma and parasitic infections [1], [2]. Eosinophils differentiate from hematopoietic stem cells via a common myeloid progenitor cell, and then an eosinophil lineage-committed progenitor marked by CD34^+^ and CD125^+^ (IL-5Rα) [3]. The cytokine IL-5 promotes the selective differentiation of eosinophils and the release of mature eosinophils from the bone marrow [1]. IL-5 has been shown to promote eosinophil survival by activating MAP kinase, Lyn tyrosine kinase and PI3 kinase signaling [4], [5]. Recently, a lineage-committed eosinophil progenitor population that gives rise exclusively to eosinophils has been identified in both murine and human bone marrow [3], [6]. This eosinophil lineage-committed progenitor population is IL-5Rα positive while the non-eosinophil lineage-committed progenitors are IL-5Rα negative [3]. Although key transcription factors have been identified that regulate eosinophil lineage commitment (e.g. C/EBP, GATA1 and PU.1), the mechanisms controlling the growth and proliferation of eosinophil progenitors remain under investigation.

MicoRNAs (miRNA) are small non-coding RNAs that regulate gene expression post-transcriptionally via either target mRNA degradation or translational inhibition. MiRNA expression profiles are distinct in different hematopoietic lineages [7]. Dysregulation of miRNA expression has been shown to affect lineage commitment of hematopoietic stem cells [8]; however, there is little known about miRNA regulation of lineage committed progenitor cells.

MiR-21 has been reported to be up-regulated in a variety of disorders with significant eosinophilia including asthma [9], ulcerative colitis [10], and eosinophilic esophagitis [11]. We have found that miR-21 was among the most up-regulated miRNAs in patients with eosinophilic esophagitis and has the highest correlation with esophageal eosinophil levels [11]. However, the function of miR-21 during eosinophil development is not known. Herein, we show that miR-21 is up-regulated during eosinophil differentiation from eosinophil progenitors and that targeted ablation of miR-21 decreases eosinophil progenitor cell growth. Eosinophil progenitor cultures derived from the miR-21^−/−^ mice have increased levels of apoptosis compared to that of miR-21^+/+^ mice. Furthermore, miR-21^−/−^ mice have decreased eosinophil colony-forming unit capacity in the bone marrow and decreased eosinophil levels in the blood. Whole-genome microarray analysis of differentially regulated genes between miR-21^+/+^ and miR-21^−/−^ eosinophil progenitor cultures identified miR-21-dependent pathways involved in the regulation of cell growth, cell cycle and immunity. Our data indicate that miR-21 can directly regulate the development of eosinophils by influencing the growth capacity of eosinophil progenitors.

## Materials and Methods

### Mice

The miR-21 gene-targeted mice were backcrossed for 5 generations into the C57BL/6 background as previously described [12]. Littermate controls were used for all experiments. All animals used were housed under specific pathogen-free conditions in accordance with institutional guidelines. The Institutional Animal Care and Use Committee of the Cincinnati Children’s Hospital Medical Center approved the use of animals in these experiments.

### Bone Marrow Derived Cell Cultures

Bone marrow cells were collected from femur and tibia of the mice, and the stem/progenitor cell enriched low-density fraction was isolated by gradient centrifugation using Histopaque 1083 (Sigma) according to the manufacturer’s protocol. The low-density fraction of bone marrow cells were cultured in IMDM with Glutamax I (Life Technologies) with 10% FBS, 100 U/mL penicillin and 100 µg/mL streptomycin supplemented with 100 ng/mL stem cell factor (SCF) and 100 ng/mL FLT-3 ligand (Peprotech) from day 0 to day 4 at a concentration of 1×10^6^/mL in 6-well plates. For eosinophil differentiation, the SCF and FLT-3 ligand were washed out and replaced with 10 ng/mL IL-5 on day 4, and the cells were cultured for an additional 10 days in the presence of IL-5 [13]. One half of the culture media was replaced with fresh media every other day. The cells were counted, and the concentration was adjusted to 1×10^6^/mL during each media change. Eosinophil maturity was assessed by FACS staining for CCR3 and Siglec-F and/or Diff-Quik staining of cytospin preparations. For neutrophil differentiation, the SCF and FLT-3 ligand were replaced with 20 ng/mL granulocyte colony-stimulating factor (G-CSF) on day 4 and the cells were cultured for an additional 6 days in the presence of G-CSF. One half of the culture media was replaced with fresh media every other day. Eosinophil and neutrophil progenitor growth was assessed by counting the cells every 2 days using a hemacytometer.

### Quantitative Assessment of miRNA Levels

Levels of miRNA expression were measured quantitatively by using the TaqMan MicroRNA Assay (Applied Biosystems) following the manufacturer’s protocol and assayed on the Applied Biosystems 7900 HT Real-Time PCR System. Normalization was performed using U6 small nuclear RNA. Relative expression was calculated using the comparative C_T_ method as previously described [14].

### Determination of Blood Eosinophil Percentage by FACS

Red blood cells were lysed from murine blood by using RBC lysis buffer (Sigma) twice for 5 min each time. The eosinophil percentage was determined by FACS staining of blood cells with FITC-conjugated anti-CCR3 (R&D systems) and PE-conjugated anti-Siglec-F (BD Biosciences). Eosinophils were the CCR3 and Siglec-F double-positive cells, as reported [15].

### Colony-forming Unit (CFU) Assay

Low-density bone marrow fractions were plated at a concentration of 1×10^5^/mL in M4230 methylcellulose media supplemented with 50 ng/mL of IL-5 for CFU-Eos assay or at a concentration of 5×10^4^/mL in M4230 methylcellulose media supplemented with 50 ng/mL G-CSF for CFU-G assay according to manufacturer’s protocols. After 8 days of incubation at 37°C and 5% CO_2_, eosinophil colonies (CFU-Eos) and neutrophil colonies (CFU-G) were counted and reported as CFU per 10^5^ cells. Cell morphology of CFU-Eos and CFU-G was confirmed by Diff-Quik staining of cytospin preparations of the colonies.

### Murine Genome-wide mRNA Microarray

The Affymetrix Mouse Gene 1.0ST array was used to compare gene expression profile between miR-21^+/+^ and miR-21^−/−^ eosinophil progenitor cultures at day 4, day 8 and day 12. Microarray data were analyzed using the GeneSpring software (Agilent Technologies). Global scaling was performed to compare genes from chip to chip, and a base set of probes was generated by requiring a minimum raw expression level of 20^th^ percentile out of all probes on the microarray. The resulting probe sets were then baseline transformed and filtered on at least 1.5-fold difference between miR-21^+/+^ and miR-21^−/−^ eosinophil progenitor cultures. Statistical significance was determined at p<0.05 with Benjamini-Hochberg false discovery rate correction. The resulting list of genes was clustered using hierarchical clustering and a heatmap was generated. Biological functional enrichment analysis was carried out using ToppGene/ToppCluster [16], [17]. The microarray data have been deposited into the Array Express database (www.ebi.ac.uk/arrayexpress) with accession number E-MEXP-3346 in compliance with minimum information about microarray experiment (MIAME) standards.

### Allergen-induced Model of Eosinophilic Esophagitis

A murine model of allergen induced eosinophilic esophagitis was performed as previously described [18]. Briefly, mice were lightly anesthetized with isofluorane, and 100 µg of *Aspergillus fumigatus* dissolved in 50 µL of saline was applied to the nares. Control mice received 50 µL of normal saline. After instillation, mice were held upright until alert. Mice were challenged 3 times per week for 3 weeks and sacrificed 18–20 hours after the last challenge. Esophageal eosinophils from esophageal paraffin tissue sections were identified with anti-MBP staining (a kind gift of Dr. Jamie Lee, Mayo Clinic, Scottsdale, AZ) and quantified by counting anti-MBP positive immunostained cells as previously described [19].

### Quantitative RT-PCR

Total RNA was reverse transcribed using the High Capacity cDNA Reverse Transcription kit (Applied Biosystems) according to manufacturer’s protocol. All primer/probe sets were obtained from Applied Biosytems. Samples were analyzed by TaqMan qRT-PCR for *Pik3r6* (Assay ID: Mm01335671_m1), *Ms4a3* (Assay ID: Mm_00460072_m1), *Psrc1* (Assay ID: Mm_00498358_m1), and *Grb7* (Assay ID: Mm_01306734_m1) and normalized to *Hprt1* (Assay ID: Mm00446968_m1). Relative expression was calculated using the comparative C_T_ method [14].

### Statistical Analysis

Statistical analyses were performed with student’s *t*-test or one-way ANOVA with Tukey post-hoc test when appropriate. Statistical significance and the p values were indicated on the figures where appropriate. P values less than 0.05 were considered statistically significant.

## Results

### Expression of miR-21 in an ex vivo Culture of Bone Marrow-derived Eosinophils

**Figure 1:**
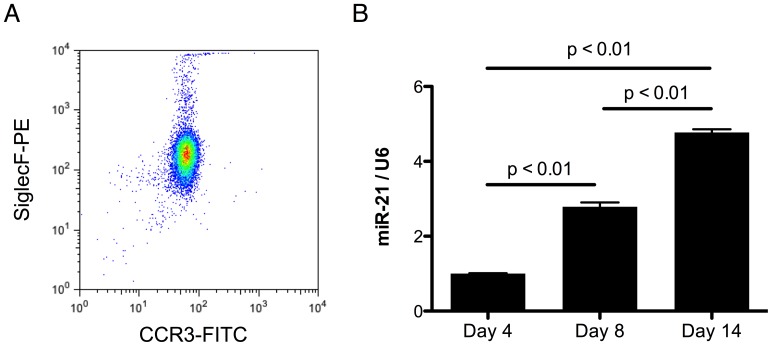
MiR-21 is induced during eosinophil differentiation. (A) Purity of cultured eosinophils at day 14. Eosinophils are identified as CCR3^+^Siglec-F^+^ cells. (B) Levels of miR-21 during the eosinophil differentiation culture determined by qPCR normalized to U6 small nuclear RNA. N = 3 per group; data are represented as mean ± S.E.M. Data are representative of 3 independent experiments.

To determine the role of miR-21 in eosinophil development and function, we utilized a murine *ex vivo* bone marrow-derived eosinophil culture model that generates of >90% eosinophils after 14 days of culture [13]. We obtained *ex vivo* bone marrow-derived eosinophils with high purity as determined by FACS staining for CCR3 and Siglec-F double-positive cells on day 14 (Fig. 1A). We found up-regulation of miR-21 during the eosinophil differentiation from day 4 to day 14 (Fig. 1B).

### Eosinophil Progenitor Growth in Eosinophil Cultures Derived from miR-21^−/−^ Mice

**Figure 2:**
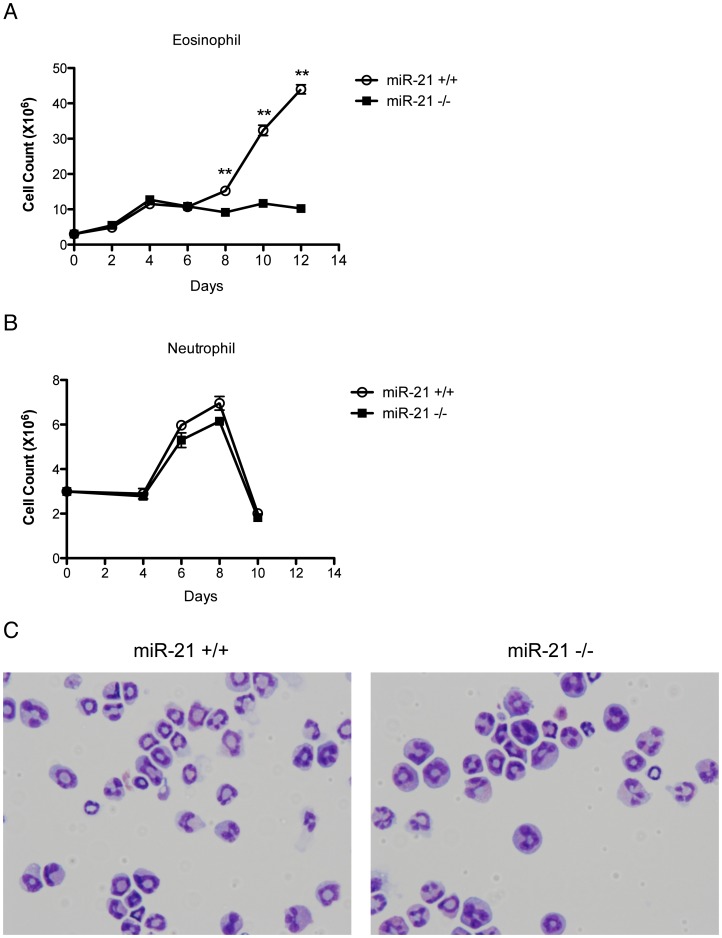
Growth of eosinophil progenitor cells from miR-21^−/−^ mice and miR-21^+/+^ controls during the ex vivo eosinophil culture. Total cell number of (A) eosinophil and (B) neutrophil cultures derived from miR-21^+/+^ and miR-21^−/−^ mice is shown. N = 6 per group; data are represented as mean ± S.E.M. **: p<0.01. (C) Morphology of miR-21^+/+^ and miR-21^−/−^ cultured eosinophils at day 12 determined by Diff-Quik staining. Data are representative of 3 independent experiments for panels A and C; data are representative of 2 independent experiments for panel B.

To determine the effect of miR-21 on eosinophil development, we cultured progenitor cells into eosinophils and noted a profound phenotype in cellular growth. The miR-21^−/−^ cells had a marked arrest in eosinophil progenitor cell growth compared to controls with the most prominent effect seen between days 8 and 12 (Fig. 2A). In comparison, neutrophil progenitor cell growth was not significantly changed (Fig. 2B). The miR-21^+/+^ and miR-21^−/−^ bone marrow derived eosinophils were morphologically indistinguishable from each other at day 12 (Fig. 2C).

### Eosinophil Cultures Derived from miR-21^−/−^ Mice have Increased Apoptosis Compared to Littermate Controls

**Figure 3:**
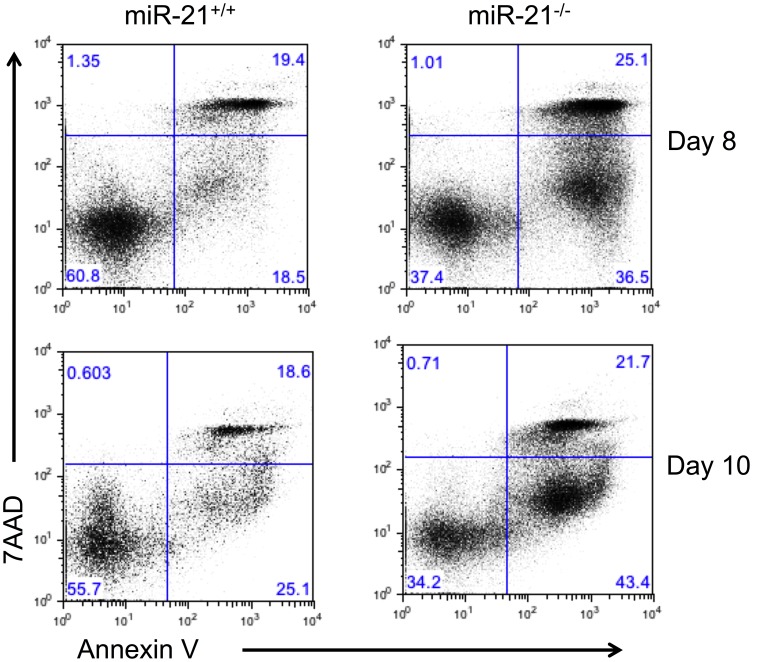
Levels of apoptosis in the eosinophil progenitor culture from the miR-21^+/+^ and miR-21^−/−^ mice. Levels of Annexin V and 7AAD staining during eosinophil differentiation culture as determined by FACS. The viable cells are AnnexinV^−^ 7AAD^−^. The early apoptotic cells are AnnexinV^+^ 7AAD^−^. The late apoptotic cells are AnnexinV^+^ 7AAD^+^. Data are representative of 3 independent experiments.

MiR-21 has been reported to be pro-proliferative and anti-apoptotic by targeting multiple tumor suppressor genes [20]–[22]. We measured the levels of apoptosis in the miR-21^−/−^ and miR-21^+/+^ eosinophil progenitor cultures by Annexin V and 7AAD staining. The viable cells are Annexin V and 7AAD double negative. The early apoptotic cells are Annexin V positive and 7AAD negative. The late apoptotic cells are Annexin V and 7AAD double positive. Compared to miR-21^+/+^ cultures, the miR-21^−/−^ eosinophil progenitor cultures have increased levels of both the Annexin V^+^7AAD^−^ population and the AnnexinV^+^7AAD^+^ population, indicative of increased levels of apoptosis in the miR-21^−/−^ cultures (Fig. 3).

### MiR-21^−/−^ Mice have Reduced Eosinophilia in the Blood and Reduced Eosinophil Colony-forming Unit Capacity in the Bone Marrow

**Figure 4:**
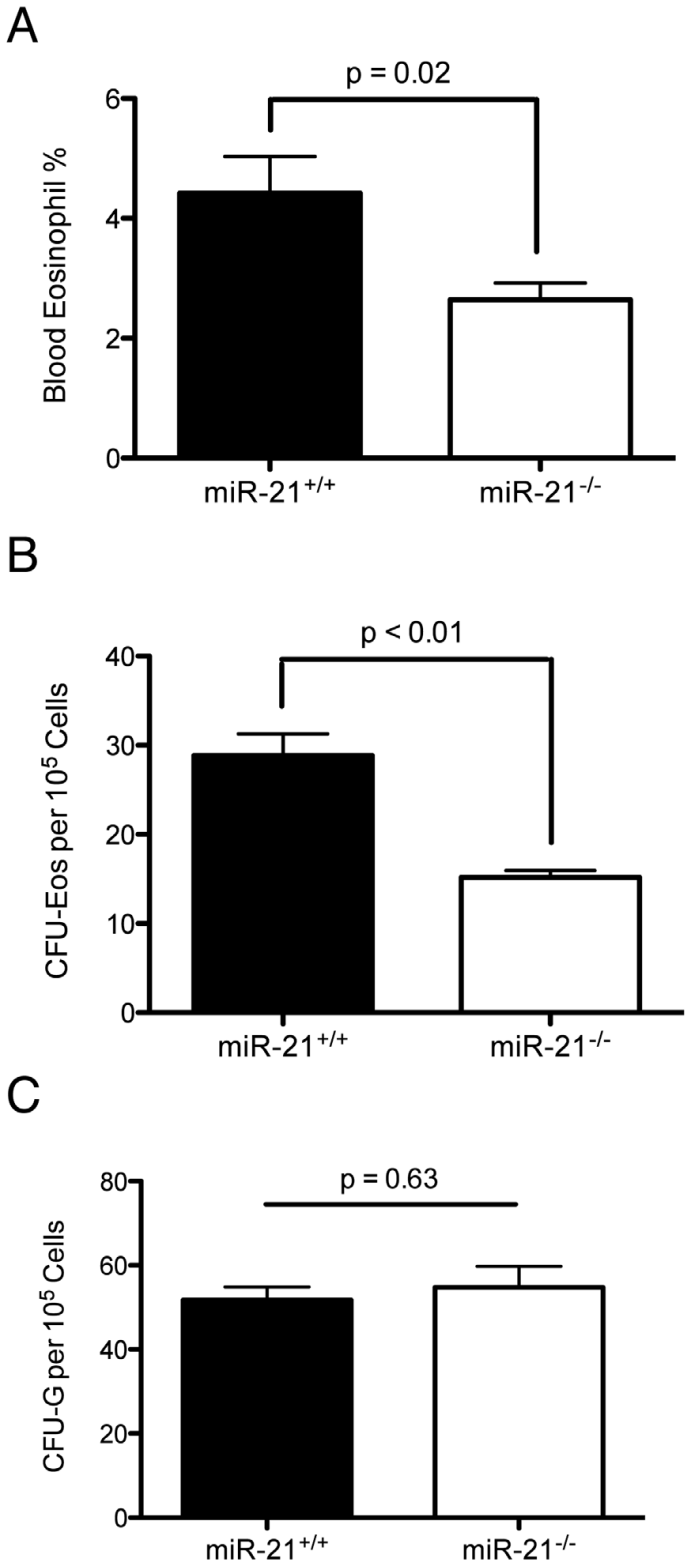
Blood eosinophil percentage and bone marrow eosinophil colony forming unit capacity in the miR-21^+/+^ and miR-21^−/−^ mice. (A) Blood eosinophil percentage from miR-21^+/+^ and miR-21^−/−^ mice determined by FACS staining for CCR3^+^Siglec-F^+^ cells; n = 9–10 mice per group. (B) Bone marrow eosinophil colony-forming unit (CFU-Eos) and (C) neutrophil colony-forming unit (CFU-G) capacity from miR-21^+/+^ and miR-21^−/−^ mice; n = 4 per group. Data are represented as mean ± S.E.M. Data are representative of 3 independent experiments.

To examine the consequences of miR-21 on eosinophil hematopoiesis *in vivo*, we determined the blood eosinophil level in the miR-21^−/−^ mice. The miR-21^−/−^ mice had a decreased blood eosinophil percentage compared to miR-21^+/+^ littermate controls (Fig. 4A). Furthermore, miR-21^−/−^ mice had decreased eosinophil colony-forming unit capacity in the bone marrow (Fig. 4B). In comparison, the neutrophil colony-forming unit capacity was unchanged (Fig. 4C), indicating an eosinophil progenitor specific defect.

### Whole-genome Microarray Analysis of Genes Differentially Regulated between miR-21^+/+^ and miR-21^−/−^ Eosinophil Progenitor Cultures

**Figure 5:**
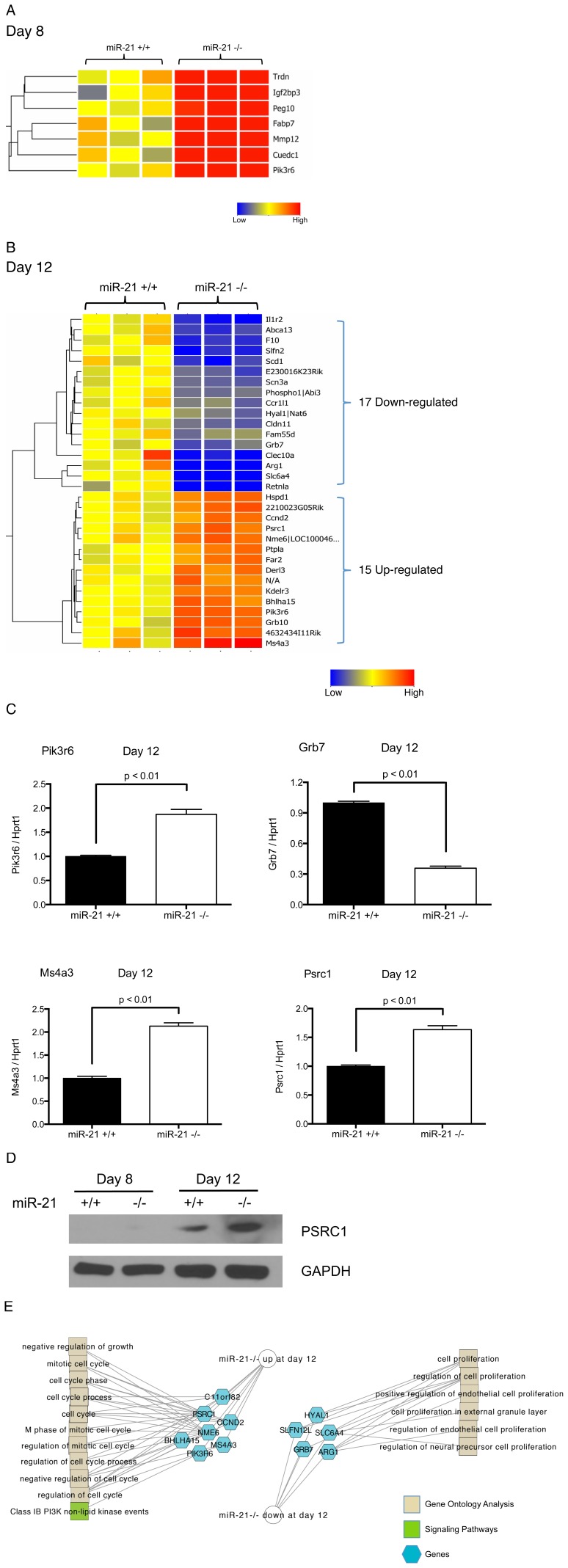
Differentially regulated genes between miR-21^+/+^ and miR-21^−/−^ eosinophil progenitor cultures at day 8 and day 12. (A) Heat map of differentially regulated genes at day 8 of the eosinophil differentiation culture. Red: up-regulated in miR-21^−/−^ eosinophil progenitor culture compared to miR-21^+/+^ eosinophil progenitor culture. (B) Heat map of differentially regulated genes at day 12 of the eosinophil differentiation culture. Red: up-regulated in miR-21^−/−^ eosinophil progenitor culture compared to miR-21^+/+^ eosinophil progenitor culture; blue: down-regulated in miR-21^−/−^ eosinophil progenitor culture compared to miR-21^+/+^ eosinophil progenitor culture. (C) Quantitative RT-PCR verification of a selected set of differentially expressed genes between miR-21^+/+^ and miR-21^−/−^ eosinophil progenitor cultures. (D) Western blot showing levels of PSRC1 in miR-21^+/+^ and miR-21^−/−^ eosinophil progenitor cultures at day 8 and day 12. GAPDH is shown as a loading control. (E) Functional enrichment analysis of differentially regulated genes in the eosinophil progenitor cultures at day 12. The networks are shown as Cytoscape graph networks generated from ToppCluster network analysis.

In order to gain insight into potential molecular mechanisms by which miR-21 regulates eosinophil development, we performed a whole genome microarray analysis at days 4, 8 and 12 of the culture of miR-21^+/+^ and miR-21^−/−^ cells. There are no differentially regulated genes at day 4 (data not shown), which is consistent with the miR-21 expression data and the lack of any observed phenotype at day 4 of the culture (Fig. 2). This suggests that the growth of stem/progenitor cells under the influence of SCF and FLT-3L is not dependent on miR-21. At day 8 of the eosinophil progenitor culture, 7 genes were up-regulated in the miR-21^−/−^ eosinophil progenitor culture compared to the miR-21^+/+^ culture and there were no down-regulated genes (Fig. 5A; Table S1). At day 12 of the eosinophil progenitor culture, there were 17 down-regulated and 15 up-regulated genes (Fig. 5B; Table S2). The up-regulated genes include *Ms4a3* and *Bhlha15,* which have known roles in inhibiting cell growth [23], [24]. The down-regulated genes include *Grb7* and *Hyal1,* which have been shown to promote cell growth [25], [26]. To validate the differentially expressed genes, we performed qRT-PCR on a selected set of differentially expressed genes including *Pik3r6*, *Ms4a3*, *Psrc1* and *Grb7* (Fig. 5C). We also measured the protein level of PSRC1 and found that PSRC1 was up-regulated in miR-21^−/−^ eosinophil progenitor cultures at day 12 compared to miR-21^+/+^ controls (Fig. 5D). Functional enrichment analysis of the differentially regulated genes identified regulation of cell growth and cell cycle as the most significantly enriched pathways at day 12 (Fig. 5E).

### MiR-21^−/−^ Mice have Decreased Esophageal Eosinophil Level in an Animal Model of Eosinophilic Esophagitis

**Figure 6:**
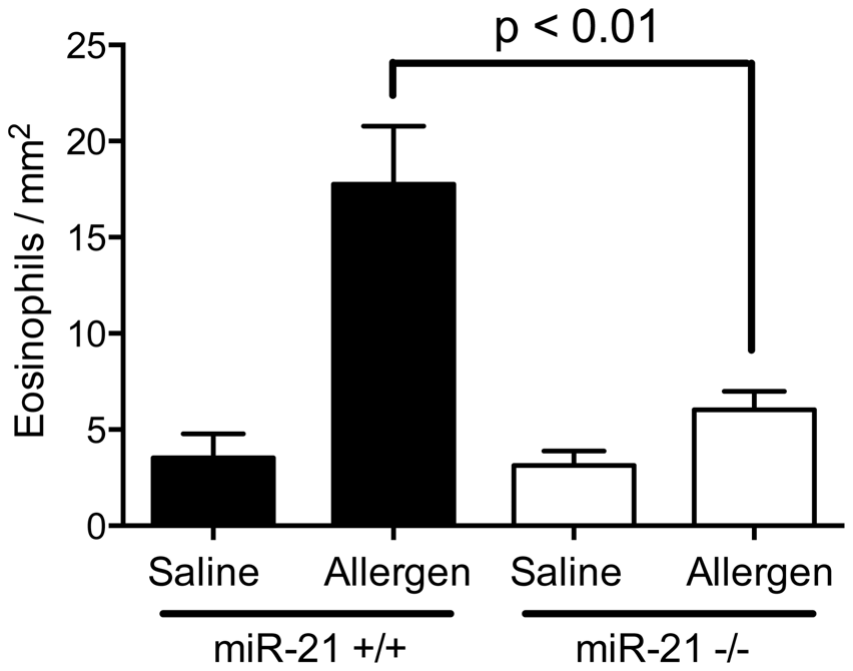
Esophageal eosinophil level in an allergen-induced experimental eosinophil esophagitis model in miR-21^+/+^ and miR-21^−/−^ mice. Eosinophil levels in the esophagus were determined by morphometric analysis following anti-MBP staining. N = 5–6 mice per group. Data are represented as mean ± S.E.M. Data are representative of 2 independent experiments.

To further examine the effect of miR-21 deficiency on eosinophil levels *in vivo*, we induced an experimental model of eosinophilic esophagitis in which eosinophil infiltration is a prominent feature of the disease and miR-21 has been shown to be the most up-regulated microRNA [11]. We found that miR-21^−/−^ mice had significantly decreased levels of allergen-induced esophageal eosinophil infiltration compared to the miR-21^+/+^ mice (Fig. 6).

## Discussion

Herein, we have shown that miR-21 is progressively up-regulated during eosinophil progenitor cell growth. Targeted ablation of miR-21 in the eosinophil progenitor cultures led to decreased growth capacity with an increased level of apoptosis *in vitro*. Whole-genome microarray analysis of miR-21^+/+^ and miR-21^−/−^ eosinophil progenitor cultures identified regulation of cell growth, cell cycle and immune response as the most significantly affected pathways by miR-21. These results demonstrate that miR-21 has an important role in regulating and fine-tuning the proliferative capacity of eosinophil progenitors. Since mature eosinophils loose their proliferative capacity and do not divide, we hypothesize that the up-regulation of miR-21 likely prevents premature loss of the proliferative potential of eosinophil progenitors.

We did not identify any differentially regulated genes at day 4 of the eosinophil progenitor culture. This supports our data that progenitor cell growth under the influence of SCF and FLT-3L is not regulated by miR-21. Between days 8 and 14, we identified 38 differentially regulated genes. While only one (*Psrc1*) is a predicted target of miR-21, it is notable that computational analysis identified an overall functional effect in the pathways (e.g. regulation of cell proliferation and cell cycle) associated with the observed phenotype and the known role of miR-21 in other systems [20], [21]. While a definitive linear relationship between miR-21 target gene and the increased apoptosis of miR-21^−/−^ eosinophil progenitors is not proven, we hypothesize that miR-21 exerts modest effects on direct targets that synergistically interact to ultimately regulate eosinophilopoeisis. Moreover, miRNAs could regulate protein translation without changing the mRNA level, which would not be identified by the genomic screen in the current study. Several other reports have shown that miR-21 targets multiple tumor suppressor and anti-proliferative genes such as *PDCD4* and *PTEN*, mostly in cancer cell lines, generally by inducing modest changes in mRNA [27]–[30]. We have previously reported that miR-21 also modestly modulates IL-12p35 and that this has a profound effect on modifying the course of immune hypersensitivity responses *in vivo* [9], [12]. We analyzed the levels of *Pdcd4* and *Pten* by qRT-PCR and found a modest up-regulation of *Pdcd4* mRNA in miR-21^−/−^ eosinophil progenitors compared to miR-21^+/+^ controls (Fig. S1). We hypothesize that the observed decreased growth capacity of the miR-21^−/−^ eosinophil progenitors could potentially be due to modest regulation of a combination of miR-21 targets, including those not identified by the genomic screen due to the modest gene expression changes. One of the up-regulated genes, *Psrc1*, is a predicted target of miR-21 based on sequence conservation and binding site potential [9]. Over-expression of *Psrc1* has been shown to suppress colony formation in lung carcinoma cells [31]. While not experimentally proven, we hypothesize that the up-regulation of *Psrc1* could contribute to the decreased growth of miR-21^−/−^ eosinophil progenitors.

MiR-21 has been known to promote cell growth in various cell types, most notable in tumor cells by targeting a variety of pro-apoptotic genes both directly and indirectly [20], [21]. Indeed, we found increased levels of apoptosis in miR-21^−/−^ eosinophil cultures compared to miR-21^+/+^ cultures. We investigated the potential eosinophil hematopoiesis defects in the miR-21^−/−^ mice *in vivo*. The miR-21^−/−^ mice have decreased eosinophils in the blood and decreased eosinophil colony-forming unit capacity in the bone marrow, in agreement with the observed phenotype in the *ex vivo* eosinophil cultures. Furthermore, in an experimental model of eosinophilic esophagitis, the miR-21^−/−^ mice have decreased esophageal eosinophils compared to miR-21^+/+^ mice. This finding is of significance since miR-21 is a top upregulated miRNA in human eosinophilic esophagitis [11], [32].

MiR-21 has been found to be over-expressed in allergic diseases with significant eosinophilia [9], [11], [32]. We have previously reported that miR-21 could regulate the immunoinflammatory responses by targeting the IL12/IFNγ pathway [9], [12]. We also found regulation of inflammatory response as one of the significantly enriched pathways represented by the differentially expressed genes in the miR-21-deficient eosinophil progenitor cultures (Fig. S2). These results suggest that miR-21 could have additional roles in regulating the immunoinflammatory responses beyond regulation of the IL12/IFNγ pathway. Given our finding that miR-21 could also affect eosinophil progenitor growth, potential therapeutic interventions targeting miR-21 might have a beneficial role in reducing the levels of eosinophilia in some circumstances.

In summary, we have identified miR-21 as a regulator of eosinophil progenitor growth. Further elucidating and understanding the roles of miR-21 in regulating the levels of eosinophils and in immunoinflammatory responses may lead to novel therapeutic options for eosinophilic disorders.

## Supporting Information

Figure S1 **Expression level of Pdcd4 in miR-21^+/+^ and miR-21^−/−^ eosinophil progenitor cultures.** Relative expression level of *Pdcd4* mRNA at day 8 and day 12 determined by qPCR normalized to *Hprt1*. N = 3 per group; data are represented as mean ± S.E.M.
(PDF)

Figure S2 **Biological function enrichment analysis of differentially regulated genes in eosinophil progenitor cultures at day 12.** Ingenuity analysis of the most significant biological functions represented by the differentially regulated genes between miR-21^+/+^ and miR-21^−/−^ eosinophil progenitor cultures.
(PDF)

Table S1 **List of differentially regulated genes between miR-21^+/+^ and miR-21^−/−^ eosinophil progenitor cultures at day 8.**
(PDF)

Table S2 **List of differentially regulated genes between miR-21^+/+^ and miR-21^−/−^ eosinophil progenitor cultures at day 12.**
(PDF)
