# Supplementary figures and images for: Targeted Ablation of miR-21 Decreases Murine Eosinophil Progenitor Cell Growth
Source: PLoS One. 2013 Mar 22;8(3):e59397. doi: 10.1371/journal.pone.0059397 (PMC3606295; doi:10.1371/journal.pone.0059397)

Figure S1

Day 8

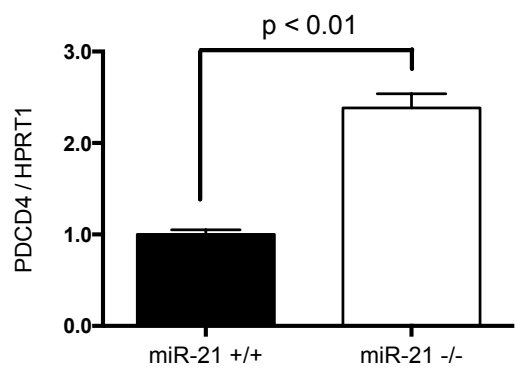

Day 12

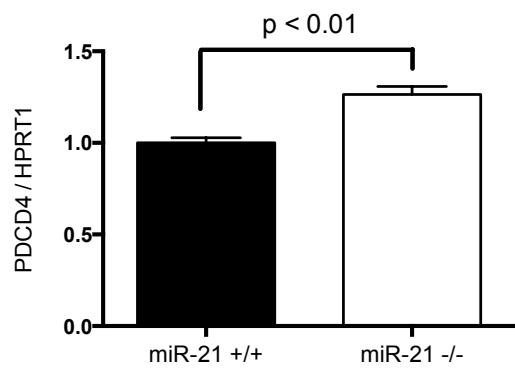

Supplement: Figure S1 — Expression level of Pdcd4 in miR-21+/+ and miR-21−/− eosinophil progenitor cultures. Relative expression level of Pdcd4 mRNA at day 8 and day 12 determined by qPCR normalized to Hprt1. N = 3 per group; data are represented as mean ± S.E.M. (PDF) [file pone.0059397.s001.pdf]

Figure S2

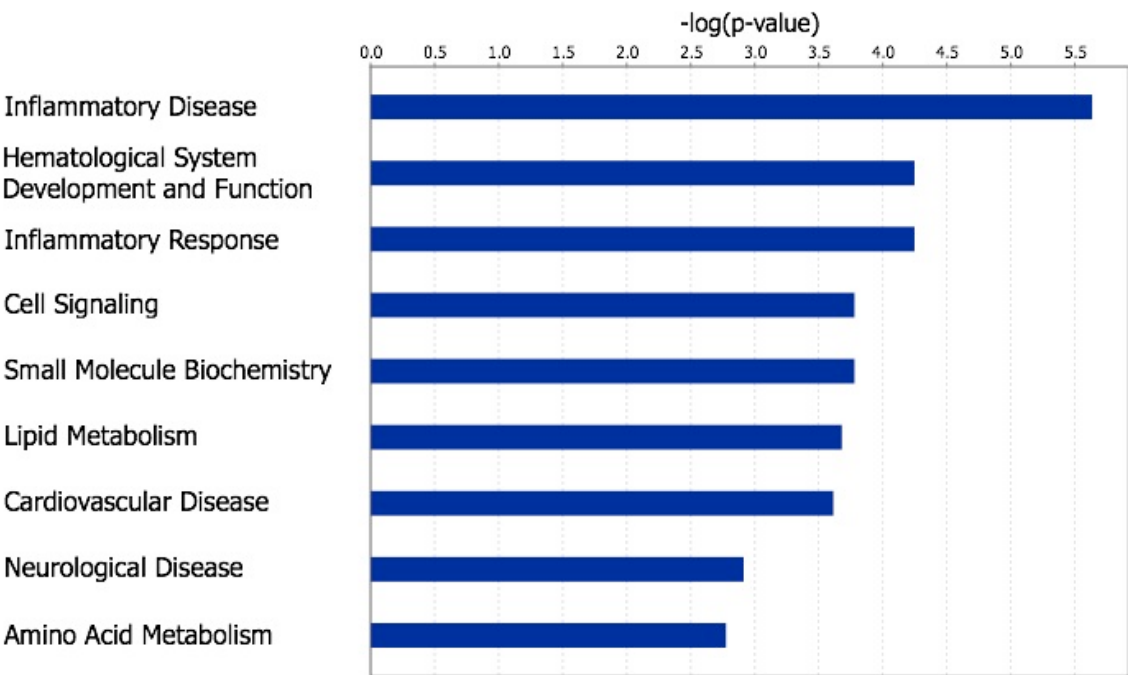

Supplement: Figure S2 — Biological function enrichment analysis of differentially regulated genes in eosinophil progenitor cultures at day 12. Ingenuity analysis of the most significant biological functions represented by the differentially regulated genes between miR-21+/+ and miR-21−/− eosinophil progenitor cultures. (PDF) [file pone.0059397.s002.pdf]
