# Supplementary material for: Targeted Ablation of miR-21 Decreases Murine Eosinophil Progenitor Cell Growth
Source: PLoS One. 2013 Mar 22;8(3):e59397. doi: 10.1371/journal.pone.0059397 (PMC3606295; doi:10.1371/journal.pone.0059397)
Supplement: Table S1 — List of differentially regulated genes between miR-21+/+ and miR-21−/− eosinophil progenitor cultures at day 8. (PDF) [file pone.0059397.s003.pdf]

Table S1

Transcript ID	Gene Symbol	Gene Description	Fold Change	Regulation
10362442	*Trdn*	triadin	1.53	Up-regulated
10363224	*Fabp7*	fatty acid binding protein 7, brain	1.73	Up-regulated
10377286	*Pik3r6*	phosphoinositide-3-kinase, regulatory subunit 6	1.79	Up-regulated
10380226	*Cuedc1*	CUE domain containing 1	2.00	Up-regulated
10536294	*Peg10*	paternally expressed 10	1.52	Up-regulated
10544610	*Igf2bp3*	insulin-like growth factor 2 mRNA binding protein 3	1.74	Up-regulated
10583056	*Mmp12*	matrix metallopeptidase 12	1.67	Up-regulated
