# Supplementary material for: Targeted Ablation of miR-21 Decreases Murine Eosinophil Progenitor Cell Growth
Source: PLoS One. 2013 Mar 22;8(3):e59397. doi: 10.1371/journal.pone.0059397 (PMC3606295; doi:10.1371/journal.pone.0059397)
Supplement: Table S2 — List of differentially regulated genes between miR-21+/+ and miR-21−/− eosinophil progenitor cultures at day 12. (PDF) [file pone.0059397.s004.pdf]

Table S2

Transcript ID	Gene Symbol	Gene Description	Fold Change	Regulation
10354732	*Hspd1*	heat shock protein 1 (chaperonin)	1.54	Up-regulated
10364093	*Derl3*	Der1-like domain family, member 3	1.53	Up-regulated
10377286	*Pik3r6*	phosphoinositide-3-kinase, regulatory subunit 6	1.67	Up-regulated
10384398	*Grb10*	growth factor receptor bound protein 10	1.76	Up-regulated
10425287	*Kdelr3*	KDEL (Lys-Asp-Glu-Leu) endoplasmic reticulum protein retention receptor 3	1.53	Up-regulated
10466224	*Ms4a3*	membrane-spanning 4-domains, subfamily A, member 3	1.88	Up-regulated
10489343	*Ptpla*	protein tyrosine phosphatase-like (proline instead of catalytic arginine), member a	1.53	Up-regulated
10495316	*Psrc1*	proline/serine-rich coiled-coil 1	1.51	Up-regulated
10527323	*Bhlha15*	basic helix-loop-helix family, member a15	1.57	Up-regulated
10542857	*Far2*	fatty acyl CoA reductase 2	1.52	Up-regulated
10548105	*Ccnd2*	cyclin D2	1.51	Up-regulated
10559818	*N/A*	N/A	1.50	Up-regulated
10565570	*4632434I11Rik*	RIKEN cDNA 4632434I11 gene	1.66	Up-regulated
10574572	*2210023G05Rik*	RIKEN cDNA 2210023G05 gene	1.58	Up-regulated
10589413	*Nme6|LOC100046163|LOC100046157*	non-metastatic cells 6, protein expressed in (nucleoside-diphosphate kinase) | similar to Nme6 protein | hypothetical protein LOC100046157	1.52	Up-regulated
10345752	*Il1r2*	interleukin 1 receptor, type II	-2.16	Down-regulated
10368343	*Arg1*	arginase, liver	-4.84	Down-regulated
10374248	*Abca13*	ATP-binding cassette, sub-family A (ABC1), member 13	-1.90	Down-regulated
10377782	*Clec10a*	C-type lectin domain family 10, member A	-2.69	Down-regulated
10378816	*Slc6a4*	solute carrier family 6 (neurotransmitter transporter,	-3.98	Down-regulated

		serotonin), member 4		
10379630	*Slfn2*	schlafen 2	-2.06	Down-regulated
10379710	*E230016K23Rik*	RIKEN cDNA E230016K23 gene	-1.64	Down-regulated
10380566	*Phospho1|Abi3*	phosphatase, orphan 1 | ABI gene family, member 3	-1.59	Down-regulated
10380927	*Grb7*	growth factor receptor bound protein 7	-1.56	Down-regulated
10436095	*Retnla*	resistin like alpha	-2.99	Down-regulated
10467979	*Scd1*	stearoyl-Coenzyme A desaturase 1	-2.00	Down-regulated
10483228	*Scn3a*	sodium channel, voltage-gated, type III, alpha	-1.55	Down-regulated
10491313	*Cldn11*	claudin 11	-1.62	Down-regulated
10570291	*F10*	coagulation factor X	-2.01	Down-regulated
10585068	*Fam55d*	family with sequence similarity 55, member D	-1.51	Down-regulated
10588691	*Hyal1|Nat6*	hyaluronoglucosaminidase 1 | N-acetyltransferase 6	-1.50	Down-regulated
10598010	*Ccr1l1*	chemokine (C-C motif) receptor 1-like 1	-1.55	Down-regulated
